# Novel Human Astroviruses: Prevalence and Association with Common Enteric Viruses in Undiagnosed Gastroenteritis Cases in Spain

**DOI:** 10.3390/v11070585

**Published:** 2019-06-27

**Authors:** Diem-Lan Vu, Aurora Sabrià, Nuria Aregall, Kristina Michl, Virginia Rodriguez Garrido, Lidia Goterris, Albert Bosch, Rosa Maria Pintó, Susana Guix

**Affiliations:** 1 Enteric Virus Laboratory, Department of Genetics, Microbiology and Statistics, University of Barcelona, 08028 Barcelona, Spain; 2 Nutrition and Food Safety Research Institute (INSA·UB), University of Barcelona, 08921 Santa Coloma de Gramenet, Spain; 3 Department of Infectious Diseases, Geneva University Hospitals, 1205 Geneva, Switzerland; 4 Microbiology Department, Hospital Universitari Vall d’Hebron, 08035 Barcelona, Spain

**Keywords:** gastroenteritis, children, novel human astrovirus, classic astrovirus, norovirus, sapovirus, rotavirus, adenovirus, epidemiology, real time RT-PCR

## Abstract

A remarkable percentage of acute gastroenteritis cases remain etiologically undiagnosed. The aim of the study was to determine the prevalence of common and emerging enteric viruses, such as novel human astroviruses, among undiagnosed samples from children with acute gastroenteritis. Epidemiological studies for novel human astroviruses are still scarce. Stool samples collected over two consecutive winter seasons (2016–2017) from children with gastroenteritis in Spain, which were negative for bacteria, rotavirus, and adenovirus by routine diagnostics were screened by real-time RT-PCR assays for the presence of classical and novel astrovirus, rotavirus, norovirus GI and GII, sapovirus, and adenovirus. Overall, 220/384 stool samples (57.3%) were positive for at least one virus. Co-infections were identified in 21% of cases. Among a total of 315 viruses identified, adenovirus was the most prevalent (*n* = 103), followed by rotavirus (*n* = 51), sapovirus (*n* = 50), classical astrovirus (*n* = 43), novel astroviruses (*n* = 42), and norovirus (*n* = 26). Novel astroviruses were present in 13.3% of virus-positive cases. Most novel astroviruses were found in children <2-year-old (30/39 children, 77%, *p* = 0.01) and were found in co-infection (66%). Only classical astroviruses demonstrated significant differences in the Cq values during mono-infections compared to co-infections. In conclusion, common enteric viruses may be frequently found in children with undiagnosed gastroenteritis, indicating the need to implement more sensitive diagnostic methods. Novel astroviruses circulate in the community and could be the cause of gastroenteritis among young children.

## 1. Introduction

Despite the large number of pathogens associated with acute gastroenteritis in children, a remarkable percentage of cases remain etiologically undiagnosed, even in developed countries. Among viral etiologies of sporadic acute gastroenteritis in children, rotavirus is historically the leading pathogen, followed by norovirus and astrovirus, although implementation of the rotavirus vaccination has decreased the incidence of rotavirus gastroenteritis in concerned countries [1]. The classical human astroviruses (HAstVs), first identified in 1975 during an outbreak of gastroenteritis in a maternity ward [2], have been identified worldwide [3] since then. Several epidemiological studies have tried to determine the exact prevalence of classical HAstVs [4], but there is a high heterogeneity among studies depending on the studied population, the diagnostic method used, the timing of sampling, and the geographic area; yet, there are currently few studies using real-time PCR assays. Overall, the prevalence of classical HAstVs in children with acute gastroenteritis ranges between 0% and 30% [4].

Next-generation sequencing technologies have allowed us to identify two novel groups of highly divergent HAstVs named MLB and VA/HMO. Sequences of novel HAstVs have been found in human stools of individuals with diarrhea [5,6,7,8], but also in asymptomatic healthy controls [9,10]. Both groups of novel HAstVs have been further divided into several genotypes: MLB1-3 for the MLB astroviruses and VA1-5 for the VA astroviruses [3,4]. To date, no definitive association between novel astroviruses and gastroenteritis has yet been established, but further epidemiologic studies have confirmed the presence of novel HAstVs worldwide [9,11,12,13,14,15,16]. Interestingly, novel HAstVs have been associated with unexpected central nervous system infections in—mostly immunocompromised—humans [17,18,19,20,21,22,23]. While novel astroviruses have been identified in every continent, data in Europe are scarce, and so far only three countries have reported a systematic screening [12,14,18].

The aim of the study was to narrow down the diagnostic gap of acute gastroenteritis in children by determining the prevalence of novel astroviruses in a pediatric population with undiagnosed gastroenteritis, and to compare their prevalence with those of common enteric viruses, using sensitive molecular diagnostic tools.

## 2. Materials and Methods

### 2.1. Patient and Sample Population

The study population was children ≤ 5-years-old presenting at outpatient clinics for symptoms of gastroenteritis of unknown etiology. Stool samples had tested negative for enteropathogenic bacteria (*Salmonella* spp., *Shigella* spp., *Yersinia* spp., *Aeromonas* spp., and *Campylobacter* spp.), rotavirus or adenovirus by routine diagnostics, including immunochromatographic tests for viruses, at the Laboratory of Microbiology of Hospital de Vall d’Hebron, which is one of the main hospitals covering the Metropolitan Area of Barcelona (Spain). No clinical data were available except the patients’ age and the date of stool collection. A total of 384 stool samples were randomly selected between January and April 2016 and between January and April 2017. This period was chosen to focus on the winter season and according to the higher incidence of viral gastroenteritis in the studied population. The study was conducted in accordance with the Declaration of Helsinki, and was approved by the Clinical Research Ethics Committee of the Hospital Universitari Vall d’Hebron (26 Feb 2016; PR(AG)32/2016).

### 2.2. Viral RNA Extraction and Real Time Polymerase- or Reverse-Transcription Polymerase Chain Reaction (Real-Time PCR or Real-Time RT-PCR) Assays for Novel Astroviruses and other Enteric Viruses

RNA was extracted from 200 μL of a 30% stool suspension using the Viasure RNA-DNA Extraction Kit (Certest Biotec, Zaragoza, Spain) following the manufacturer’s instruction. Real-time RT-PCR specific assays for HAstV-MLB and HAstV-VA were performed using primers and probes previously published [14]. The standard curves and positive controls for MLB1, MLB2, MLB2-3, VA1, VA2, VA3, VA4 real-time RT-PCR assays were obtained using plasmid constructs kindly provided by Dr. S. Cordey from the Geneva University Hospitals. Standard curves were constructed based on 10-fold serial dilutions of the corresponding plasmid and analyzed in duplicate, for monoplex and duplex assays (MLB1-MLB2/3, VA1-VA2, and VA3-VA4). Table 1 was adapted from Reference [14] and provides the results of the monoplex and duplex validation assays. Real-time RT-PCR was performed using the Kapa Probe Fast Universal One-Step real-time RT-PCR Master Mix (Kapa Biosystems, Wilmington, DE, US) following the manufacturer’s instructions, on a Stratagene Mx3000P (Thermofischer, Waltham, MA, US) and a CFX96 Touch™ Real-Time PCR Detection System (Bio-Rad, Hercules, CA, US). Fifteen µL of the real-time RT-PCR master mix were mixed with 5 µL of extracted RNA. The reaction conditions were as follows: 42 °C for 15 min, 95 °C for 5 min, then 40 cycles of 95 °C for 3 s, 55 °C for 20 s, 72 °C for 10 s. Samples positive by MLB2-3 real-time RT-PCR assay were further screened with the specific MLB2 real-time RT-PCR assay to confirm the genotype [14].

We also tested the presence of other enteric viruses in stool samples by real-time RT-PCR assays targeting the classical HAstVs, sapovirus, rotavirus, adenovirus, and noroviruses GI and GII, which were available for the 2017 study period only, using available commercial kits (VIASURE Astrovirus Real Time PCR Detection Kit, VIASURE Sapovirus Real Time PCR Detection Kit, VIASURE Adenovirus Real Time PCR Detection Kit, VIASURE Norovirus GI Real Time PCR Detection Kit, VIASURE Norovirus GII Real Time PCR Detection Kit, and VIASURE Rotavirus Real Time PCR Detection Kit from Certest Biotec; and RIDA^®^GENE Viral Stool Panel II from R-Biopharm).

### 2.3. Statistical Analyses

The Kruskal-Wallis test was used to compare continuous variables and Fischer exact test was used to compare categorical variables. The non-parametric equality-of-medians test (Pearson chi2 test with continuity correction) was used to compare the proportion of Cq value above and under the median, respectively, according to mono- or poly-infection status. *p* < 0.05 was considered statistically significant. Statistics were performed by Stata /IC 13.1 (StataCorp, College Station, TX, USA). 

## 3. Results

### 3.1. More than Half of Samples Undiagnosed by Routine Screening are Positive by Real-Time PCR- or RT-PCR Assays

During the whole study period, 384 undiagnosed stool samples (*n* = 197 in 2016 and *n* = 187 in 2017) were analyzed. This selection of samples represents 24.2% of the total amount of samples from outpatient children ≤5-years-old with gastroenteritis symptoms, and to 33.1% of undiagnosed specimens. The overall proportion of undiagnosed samples during 2016 and 2017 was 77.7% (73.0% during the studied period). The mean patients’ age was 1.6 years old. Of all analyzed samples, 101/197 (51%) and 119/187 (64%) samples were identified positive for at least one virus screened by the real-time PCR or real-time RT-PCR assays. The proportion of samples that contained more than one viral target was 13% and 28%, for 2016 and 2017, respectively (average of 21% for the total number of tested samples). This led to a total of 133 and 182 distinct viruses identified in 2016 and 2017, respectively. In 2016, adenovirus was the most prevalent (47/197, 23.9%), followed by rotavirus and sapovirus (23/197 each, 11.7%), novel HAstVs (22/197, 11.2%), and classical HAstVs (15/197, 7.6%). In 2017, adenovirus remained the most prevalent (56/187, 29.9%), followed by rotavirus and classical HAstVs (28/187 each, 15%), sapovirus (27/187, 14.4%), norovirus GII (22/187, 11.8%), and novel HAstVs (17/187, 9.1%). Norovirus GI was found in only 4/187 samples (2.1%).

### 3.2. Diverse Novel HAstVs are Circulating in the Pa]ediatric Population, often in Children under 2 Years Old and in Co-Infection with other Enteric Viruses

Prevalences of novel HAstVs were 11.2% (5.6% of HAstV-MLB and 5.6% of HAstV-VA) and 9.1% (3.2% of HAstV-MLB and 5.9% of HAstV-VA) in 2016 and 2017, respectively. Among HAstV-MLB and HAstV-VA, we found 10/3 MLB1 and 1/3 MLB2, 5/6 VA1, 3/2 VA2, 5/3 VA3, and 1/0 VA4 in 2016/2017, respectively. Of note, two samples were positive for several HAstVs-VA in 2016 (one double co-infection VA1-VA2 and one triple co-infection VA1-VA2-VA3), leading to a total number of 25 novel HAstVs identified in 2016; nevertheless, these samples were considered as mono-infected in the rest of the study.

**Figure 1:**
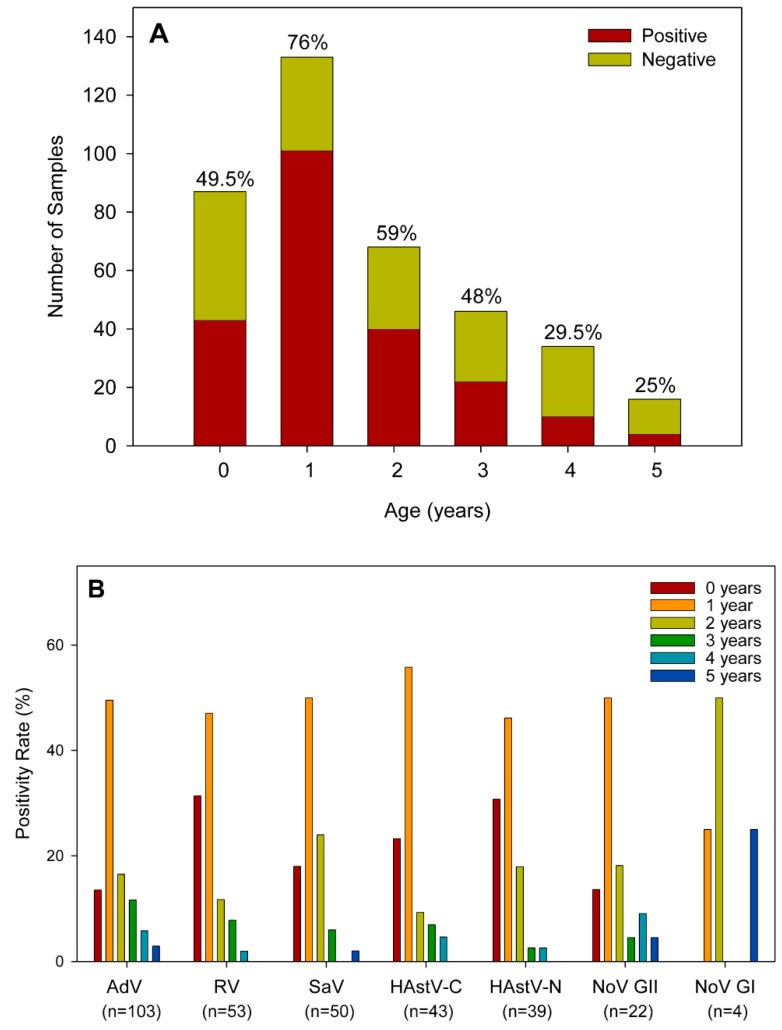
(**A**) Distribution of positive samples for any viral target by age groups. Percentages above each bar indicate the positivity rate in each group category. (**B**) Percentage distribution of positive samples by age group for each viral target. The number in brackets indicates the total number of positive samples for each virus. AdV: adenovirus; RV: rotavirus; SaV: sapovirus; HAstV-C: classic human astrovirus; HAstV-N: novel human astrovirus; NoV: norovirus: GI: genogroup I; GII: genogroup II.

According to patients’ age, 84% (184/220) positive samples for any virus were identified among children 0–3 years old (Figure 1A). Novel HAstV prevalence was highest in children under 2 years old (30/39 positive samples, 77%; *p* = 0.01) (Figure 1B). For all viral targets except for NoV GI, for which the number of positive samples was low, the age group with a higher positivity rate was the group of one-year old children. For rotavirus, classic HAstV, and novel HAstV, this group was followed by children younger than one year of age. For adenovirus, sapovirus, and norovirus GII, the second age group most affected was children 2 years-old. These same viral targets were also the ones occasionally isolated in children 5 years of age.

**Figure 2:**
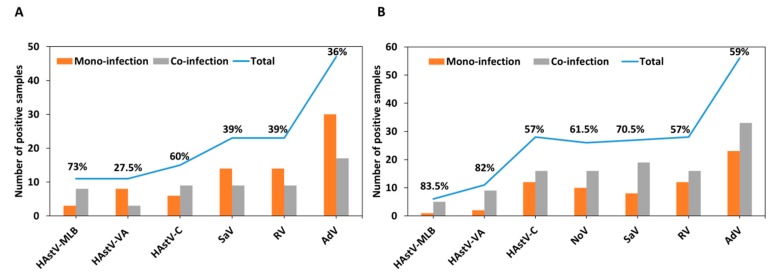
Number of cases identified in mono- and co-infections, respectively, for each virus in 2016 (**A**) and 2017 (**B**). Percentages indicate the proportion of co-infections. HAstV-MLB: MLB human astrovirus; HAstV-VA: VA human astrovirus HAstV-C: classic human astrovirus; NoV: norovirus: GI: genogroup I; GII: genogroup II; SaV: sapovirus; RV: rotavirus; AdV: adenovirus.

As for most viruses, novel HAstVs were frequently found in co-infection: 76.4% of HAstV-MLB and 54.5% of HAstV-VA positive samples (i.e., 66% of novel HAstV) were also positive for other enteric viruses. Globally, we identified a higher rate of co-infection during 2017 than during the 2016 study period (62.6% vs 42.3%, respectively). In comparison, co-infection was found in 48.5% of adenovirus, 49% of rotavirus, 56% of sapovirus, 58% of classical HAstVs, 63.6% of norovirus GII, and 50% of norovirus GI cases. Figure 2 shows the number of samples and proportion of mono- and co-infections identified per virus for each year analyzed. Overall, HAstV-MLB was identified in co-infection with adenovirus (*n* = 8), rotavirus (*n* = 5), sapovirus (*n* = 4), and classical HAstV (*n* = 1). HAstV-VA was identified in co-infection with classical HAstVs (*n* = 6), adenovirus (*n* = 5), norovirus GII (*n* = 2), and rotavirus (*n* = 1). HAstV-MLB and HAstV-VA were found in triple co-infection in three and two samples, respectively, and HAstV-MLB was also found in one quadruple co-infection (Table S1). There was no case of co-infections between HAstV-MLB and HAstV-VA genotypes. 

**Figure 3:**
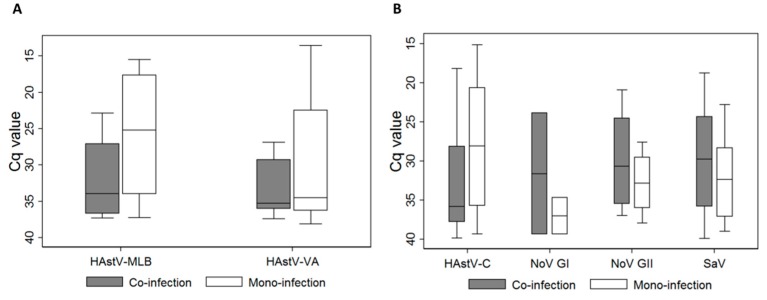
Median Cq values and confidence intervals for novel astroviruses (**A**) and other enteric viruses (**B**) according to mono- or co-infection status. Rotavirus and adenovirus were excluded from the analysis, as samples positive for these viruses by immunochromatography were excluded from the study. There was a statistical difference when comparing the Cq value during co-infection and mono-infection for classical HAstVs (*p* = 0.042 by Pearson chi2 test with continuity correction). HAstV-MLB: MLB human astrovirus; HAstV-VA: VA human astrovirus HAstV-C: classic human astrovirus; NoV: norovirus: GI: genogroup I; GII: genogroup II; SaV: sapovirus.

Mean global Cq values for novel HAstVs were 30.7 ± 6.7 for HAstV-MLB and 31.7 ± 6.4 for HAstV-VA. Figure 3 shows the median Cq values and confidence intervals for novel astroviruses (Figure 3A) and other enteric viruses (Figure 3B) during mono- and co-infections, respectively. There was no significant statistical difference in the Cq value during co-infection compared to mono-infection for any virus, except for classical HAstVs (*p* = 0.042 by Pearson Chi2 test with continuity correction).

## 4. Discussion

In the present study we demonstrated that viral sequences belonging to one or several viruses could be detected in 50%–65% of cases of gastroenteritis of unknown etiology from an outpatient pediatric population, when screened by sensitive molecular assays. These results suggest that the wide use of molecular assays on a routine basis might reduce the proportion of undiagnosed specimens from children with gastroenteritis, which is currently ~75%, down to ~30%.

While most epidemiological studies on enteric viruses in children identified rotavirus as the first viral cause of acute sporadic gastroenteritis [24,25,26,27,28], we found a higher positivity rate for adenovirus, which could be biased on the fact that positive samples for rotavirus and adenovirus by immunochromatography were excluded from the analyses. Yet, two other recent studies also identified higher prevalence of adenovirus than rotavirus infection among a cohort of children with gastroenteritis using real-time PCR assays [29,30], including one study that focused on the post rotavirus vaccine era [30]. In addition, the possibility that the real-time PCR kit used for adenoviruses detects as well other serotypes different from 40 and 41 with a lower efficiency cannot be completely ruled out. As for rotavirus, despite that the prevalence reported in this study (11.7% in 2016 and 15% in 2017) does not include samples that were positive by immunochromatographic tests, it is still remarkable, and could be partially explained by the fact that the rotavirus vaccination coverage in Spain varies between 17%–50%, depending on the year and the area studied [31].

We included the screening for norovirus only during the 2017 study period. We found that norovirus accounted for 14% of the virus-positive cases; similar to what was recently described by Martinez Ascona et al. [26]. Norovirus screening contributed to an increase of 5% (10/187) of samples with a microbiological diagnosis. The proportion of samples that remained undiagnosed decreased from 48.7% in 2016 down to 36.4% in 2017. Genogroup II was predominant, accounting for 85% of identified norovirus, as frequently described in sporadic cases of gastroenteritis [24,25,32].

While most epidemiological studies on classical HAstVs found a 2%–10% positivity rate during acute gastroenteritis [4,13,33], we found an overall prevalence of 11.5%. An 18% positivity rate was also identified by real-time RT-PCR methods in an Italian study [34], and up to 35% in a large cohort of children among three continents [35]. In accordance with the fluctuation for classic HAstV infections according to the year of sampling reported by several studies [3], the observed prevalence for classic HAstV in our study varied between 7.6% in 2016 and 15% in 2017.

Finally, in addition to the most common well-characterized enteric viruses implicated in viral gastroenteritis, we identified for the first time in Spain, a significant positivity rate for novel astroviruses, with a prevalence of 11.2% in 2016 and 9.1% in 2017. Our overall 10% prevalence is by far one of the highest described to date. While other studies found a positivity rate between 0%–6% [4,11,12,13,14], a rate similar to ours was found only in Japan [15]. Like in other recent studies [11,12,15], we found HAstV-MLB1 to be the most prevalent one. We detected no HAstV-MLB3 genotypes; interestingly, this genotype was strongly associated with asymptomatic infections in a previous study [9]. Our overall high positivity can be explained by the highly sensitive real-time RT-PCR assay we employed [36], and also by the strict sample selection criteria: absence of a bacterial cause, symptomatic children ≤5 years old, outpatients and winter season. While the winter season is usually associated with peaks of acute viral gastroenteritis [25,26,37], we cannot exclude that novel HAstV may have a different (or no) seasonal pattern. Yet, in Germany, two peaks of HAstV detection were observed, between February and May and between September and December [12], while in Thailand, the pattern of seasonal distribution varied from one year to another [11].

For all viral targets included in the study, the proportion of samples presenting co-infections was high, ranging between 27.5% and 83.5%. A similar high rate of co-infections during viral gastroenteritis was also found in other studies using real-time RT-PCR techniques [34], including classical HAstVs (77%–80%) [34,35]. Khamrin et al. also found overall 46% of co-infections among novel HAstV positive cases [15]. This highlights that viral detection should be interpreted with caution in the era of highly-sensitive molecular diagnostic tests (which detect viral sequences and not antigens). When assessing whether viral load, estimated by the Cq value, could correlate with occurrence of mono- or co-infections, we only found a statistical difference in the mean Cq value for the classical HAstVs. This difference was not statistically significant for the MLB or VA HAstVs, but it is possible that the sample size of the novel HAstVs was too small. Yet, even in the presence of a true viral co-infection, the question is not as simple as to determine which pathogen is responsible for the disease, but what is the respective contribution of each pathogen identified; specific interactions during viral co-infections should be further investigated by basic virological studies. Of note, for instance, using an immunodeficient mouse model, astroviruses have been recently shown to be elements of the virome, which can protect mice against murine norovirus and rotavirus infections [38].

In conclusion, our study provides additional information on novel HAstVs epidemiology in Europe. As in Germany, UK, and Switzerland, novel HAstVs are circulating in Spain among children with symptoms of gastroenteritis. In light of the high rate of co-infections, it remains to be determined whether novel HAstVs are actual etiologic agents of acute gastroenteritis or only bystanders and/or helpers versus controllers of other pathogens. Yet, our findings demonstrating the presence of one or more viral agents in more than half of undiagnosed gastroenteritis by routine diagnostics in children not only have clinical implications, such as the possible unnecessary use of antibiotics, but also give clues for future research studies on viral interactions and their respective contribution to gastroenteritis symptoms.

## Figures and Tables

**Table 1:** Nucleotide sequences of the primers and probes used and standard curves parameters of the novel HAstV real-time RT-PCR assays.

							Standard Curve (10-Fold Serial Dilutions)					
							Monoplex			Duplex		
Assay	Viruses Detected	Target Region (ORF)/Amplicon size (nt)	Fwd primer (5’-3’) ^1^	Probe (P) (5’-3’) ^1^	Rev Primer (5’-3’) ^1^	Final [uM] Fwd/Rev/P	Slope	Intercept	R^2^	Slope	Intercept	R^2^
**MLB1**	MLB1	ORF2/68	GGTCTTGGAGCYCGAATTC	FAM—TAGRGTTGGTTCAAATCT—MGBNFQ	CGCTGTTTAATGCGCCAAA	0.6/0.6/0.25	−4.02	43.06	0.99	−3.5	40.10	0.97
**MLB2–3**	MLB2-MLB3	ORF1b/71	CCGAGCTCTTAGTGATGCTAGCT	FAM—CGCTTCACTCGGAGAC—MGBNFQ	CACCCCTCCAAATGTACTCCAA	0.6/0.6/0.2	−3.34	44.29	0.99	−3.19	44.69	0.99
**VA1**	VA1/HMO-C/SG/PS/UK1	ORF2/66	CCATCAGCAGTTACYGGGTCTGT	FAM—TTTCCGCATATCCC—MGBNFQ	CGTGGCTCCAGGTGAYTGT	0.6/0.6/0.2	−2.72	35.24	0.96	−2.83	36.03	0.97
**VA2**	VA2/HMO-A	ORF2/67	CAGGGCCTGAATTACAAATTTCA	FAM—CATTTATGCATCCTGCTTT—MGBNFQ	GTGCCATCATTTGGCTCTTTC	0.9/0.9/0.25	−3.15	41.24	0.99	−2.71	37.96	0.98
**VA3**	VA3/HMO-B	ORF1b/67	TTCCAGGCATTTGAGTTTGCT	FAM—TTGAATCCGGATAAAAC—MGBNFQ	CCCATCCTTCTCTCAGTTCATCA	0.6/0.6/0.25	−3.16	39.71	0.98	−3.25	40.39	0.97
**VA4**	VA4	ORF2/62	GATCCATGTATCGTGCATCGTT	FAM—AACCTTACACAGTCCCCGG—MGBNFQ	GCCCCCCCAAGATGTTG	0.9/0.9/0.25	−3.17	37.94	0.98	−2.5	35.83	0.95

^1^ Primers and probes were previously published [14].

## Supplementary Materials

The following are available online at https://www.mdpi.com/1999-4915/11/7/585/s1, Table S1: Number of co- infections identified for novel HAstVs.
