# Supplementary material for: Novel Human Astroviruses: Prevalence and Association with Common Enteric Viruses in Undiagnosed Gastroenteritis Cases in Spain
Source: Viruses. 2019 Jun 27;11(7):585. doi: 10.3390/v11070585 (PMC6669616; doi:10.3390/v11070585)
Supplement: Supplementary file 1 [file viruses-11-00585-s001.pdf]

Table S1. Number of co- infections identified for novel HAstVs.

	HAstV-MLB	HAstV-VA
**Double infections**		
**Adenovirus**	4	4
**Classic HAstV**	1	5
**Rotavirus**	3	0
**Sapovirus**	1	0
**Norovirus**	0	1
**Triple infections**		
**Adenovirus and Rotavirus**	1	1
**Adenovirus and Sapovirus**	2	0
**Classic HAstV and Norovirus**	0	1
**Quadruple infections**		
**Adenovirus, Rotavirus and Sapovirus**	1	0
